# Retinal detachment complicating ocular coloboma

**DOI:** 10.1038/s41433-026-04806-5

**Published:** 2026-08-21

**Authors:** Aijing Wang, Martin P. Snead

**Affiliations:** 1 University Hospital of Wales, Heath Park Way, Cardiff, UK; 2 Vitreoretinal Research Group, John van Geest Centre for Brain Repair, University of Cambridge, Cambridge, UK

**Keywords:** Health care, Genetics

## Abstract

Choroidal, optic disc and crystalline lens colobomas are rare ophthalmological conditions, but they can (either directly or by association) present with retinal detachments that are difficult to manage. Due to their rarity and variability of presentation, evidence of different approaches to treatment strategies is mainly found in case reports or small case series. This article reviews the basic pathogenesis of these conditions and the authors’ experience in how various treatment options address the underlying mechanisms. Sub-categories of retinal detachments complicating choroidal coloboma and management strategies are discussed—principally those where retinal detachment is secondary to choroidal coloboma and those where the management of retinal detachment is complicated by the co-existence of choroidal coloboma. The pathogenesis, natural history and management strategies for retinal detachment associated with optic disc pit are reviewed together with the additional challenges posed by lens and zonular coloboma in the management of retinal detachment.

## Introduction

Choroidal, optic disc and crystalline lens colobomas are rare ophthalmological conditions but they can (either directly or by association) present with retinal detachments that are difficult to manage. Due to their rarity and variability of presentation, evidence of different approaches to treatment strategies is mainly found in case reports or small case series. This article reviews the basic pathogenesis of these conditions and the authors’ experience in how various treatment options address the underlying mechanisms. Sub-categories of retinal detachment complicating choroidal coloboma and management strategies are discussed—principally those where retinal detachment is secondary to choroidal coloboma and those where the management of retinal detachment is complicated by the co-existence of choroidal coloboma. The pathogenesis, natural history and management strategies for retinal detachment associated with optic disc pit are reviewed. Whilst not ‘true’ colobomas, retinal detachment may also be associated with agenesis of the zonule of the crystalline lens, with the additional challenges this may pose in the management of retinal detachment presenting in such patients.

### Choroidal coloboma and retinal detachment

**Fig. 1:**
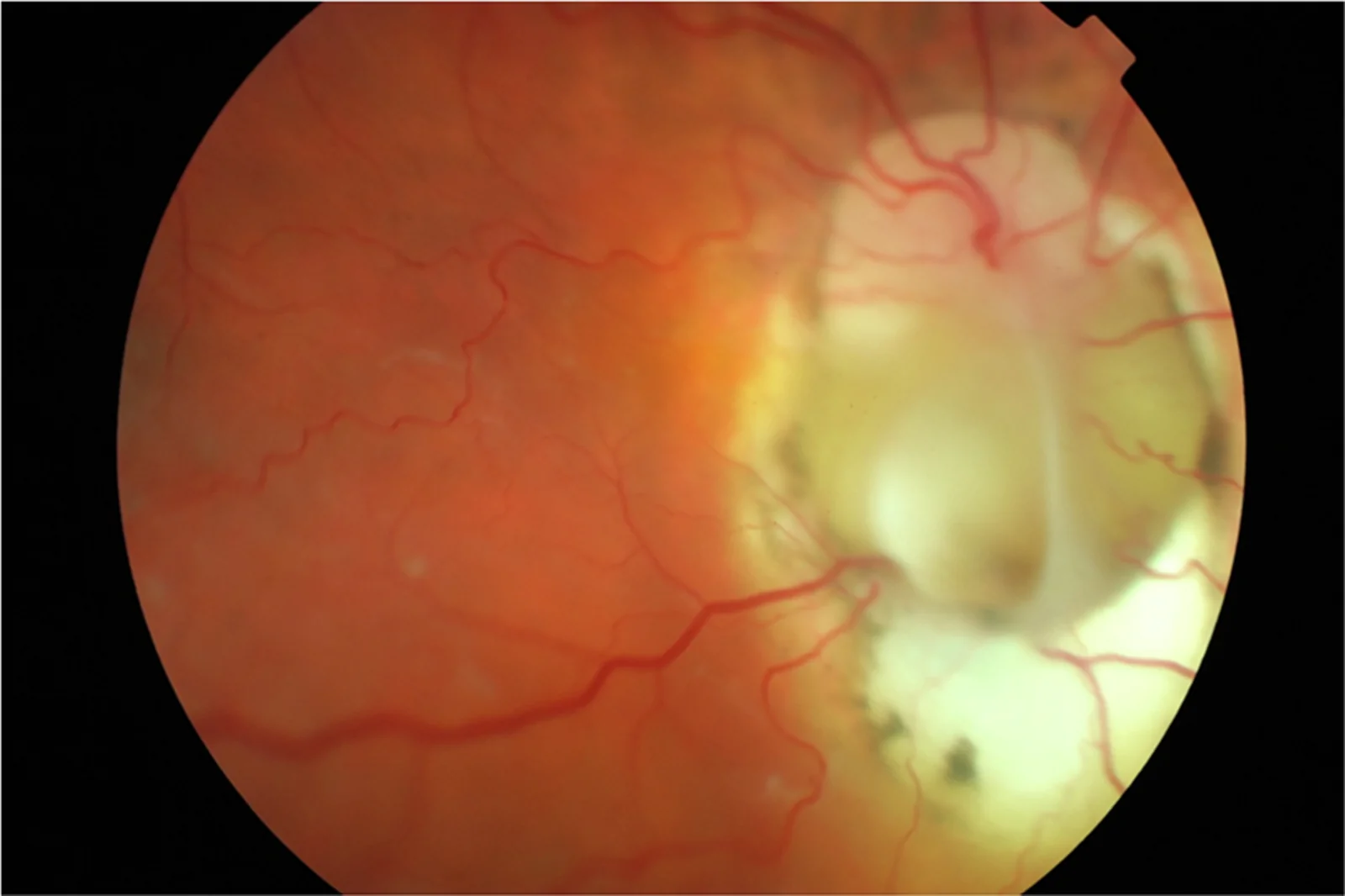
Optic disc coloboma associated with macula-off retinal detachment in CHARGE syndrome. Reproduced from Snead MP. Retinal detachment in childhood. In: Lyons CJ & Lambert SR (eds). Taylor and Hoyt’s Pediatric Ophthalmology and Strabismus. 2023; 6th edn. Elsevier Saunders. pp 613-629.

Choroidal coloboma is a congenital defect caused by defective closure of the embryonal fissure. The optic vesicle develops as a diverticulum from the lateral aspect of the forebrain and then becomes invaginated to form the double-layered optic cup. The inferior edge of the optic cup is deficient and is continuous with a groove on the inferior aspect of the optic stalk called the optic or choroidal fissure. This fissure allows vascular mesenchyme to enter the optic cup to supply the lens vesicle and is normally closed by the seventh week of embryonic development. Failure of this fissure to close completely results in a coloboma, which may include the pupil, ciliary body, choroid or optic nerve [1]. Choroidal coloboma is rare, with an estimation of 14-19:10 000 in the general population [2, 3] and has been reported in association with many different systemic conditions [4] (Table 1 and Fig. 1).

**Table 1:** Genetic conditions associated with colobomas, taken from the American Association for Paediatric Ophthalmology and Strabismus (AAPOS) [4].

Genetic conditions associated with colobomas
CHARGE association
Trisomy 13 or 18
Cat eye syndrome
Jacobsen syndrome
Goldenhar syndrome
Meckel syndrome
Morning glory anomaly
Rubinstein-Taybi syndrome
Warburg syndrome
SOX2-related eye disorders
Joubert syndrome

**Fig. 2:**
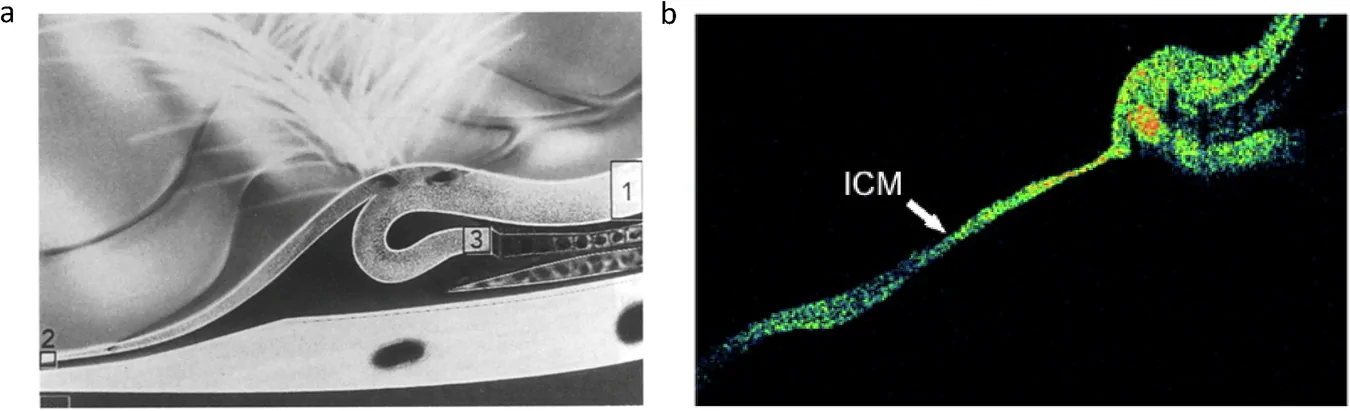
Schematic and OCT illustration of coloboma margin reproduced with permission from Schubert and Gopal. Schematic illustration of paediatric coloboma histopathology (**a**) and OCT image (**b**) showing schisis-like split of the neurosensory retina at the coloboma margin with inner layer continuing as intercalary membrane and outer layer reverting backward to join RPE. 2a: 1= extracolobomatous retina; 2= intercalary membrane; 3= reversal and duplication; reproduced from Schubert HD. Schisis-like rhegmatogenous retinal detachment associated with choroidal colobomas. Graefe’s Arch Clin Exp Ophthalmol. 1995 Feb 1;233(2):74–9. 2b reproduced from Gopal L, Khan B, Jain S, Prakash VS. A Clinical and Optical Coherence Tomography Study of the Margins of Choroidal Colobomas. Ophthalmology. 2007 Mar 1;114(3):571–80.

Schubert and Green [2] carried out important comparative studies investigating the pathology of choroidal coloboma in paediatric and adult specimens. Their studies showed a schisis-like split of the neurosensory retina at the coloboma margin, with the inner layer continuing as an intercalary membrane (ICM) and the outer layer reverting backward to join the retinal pigment epithelium (RPE) (Fig. 2).

In the paediatric specimens, the outer layer reversion was to a more laterally displaced RPE with inverted photoreceptors, observing that the glial tissue in the inner layer neither bridged the gap nor contributed to tight junctions. Based on this observation, any break in ICM was considered more likely to progress to detachment—a so-called ‘locus minoris resistentiae’ [2] (Fig. 2).

In contrast, adult specimens exhibited a more abrupt termination of the RPE and choroid at the coloboma border, both being displaced centrally with thickening and hyperplasia, thus eliminating the point of reversal and locus minoris resistentiae seen in the paediatric specimens [2].

Retinal detachment associated with choroidal colobomas has been estimated to account for 0.5% of paediatric retinal detachments [5]. In a 25-year study of 343 children with posterior segment coloboma, 5% were found to have suffered retinal detachment at a mean age of 6 years [6]. In common with other causes of retinal detachment in children (and aside from the surgical challenges), the prognosis may be further compromised by delayed presentation.

The incidence of retinal detachment complicating colobomas in adults varies widely in different series (2.4% to 47.5%) (ref. 1) but may be classified into three categories [7]:

### Type I

Retinal detachment unrelated to the coloboma

### Type IIA

Retinal detachment confined to the intercalary membrane and confined within the border of the coloboma

### Type IIB

Retinal detachment involving both the coloboma and extra-coloboma tissue [7].

In a study of 51 eyes with retinal detachment complicating choroidal coloboma, Hussain et al. identified retinal breaks within the bed of the coloboma in 26.7%, retinal breaks outside the coloboma in 33.3%, and in 13.3% breaks were identified both inside and outside the coloboma region [8]. Importantly, and highlighting one of the many surgical challenges of managing such patients, the location of the break was not identified in a quarter of all eyes [8].

A study of 30 eyes by Gopal et al. [9] concluded that retinal detachment associated with choroidal coloboma was most frequently secondary to breaks within the thin ICM, providing a conduit to the sub-retinal space. OCT scans were consistent with the post-mortem pathological studies by Schubert (Fig. 2) and highlighted that the communication sinus may be very localised in some cases [9].

Management of retinal detachment complicating ocular coloboma can be challenging in presentation, examination and surgical repair since they are frequently associated with other ocular and/or systemic comorbidities. If the pre-detachment vision is compromised for any of these reasons, presentation may be delayed (particularly in paediatric patients), thereby increasing the risk of secondary proliferative vitreoretinopathy and further complicating management.

**Fig. 3:**
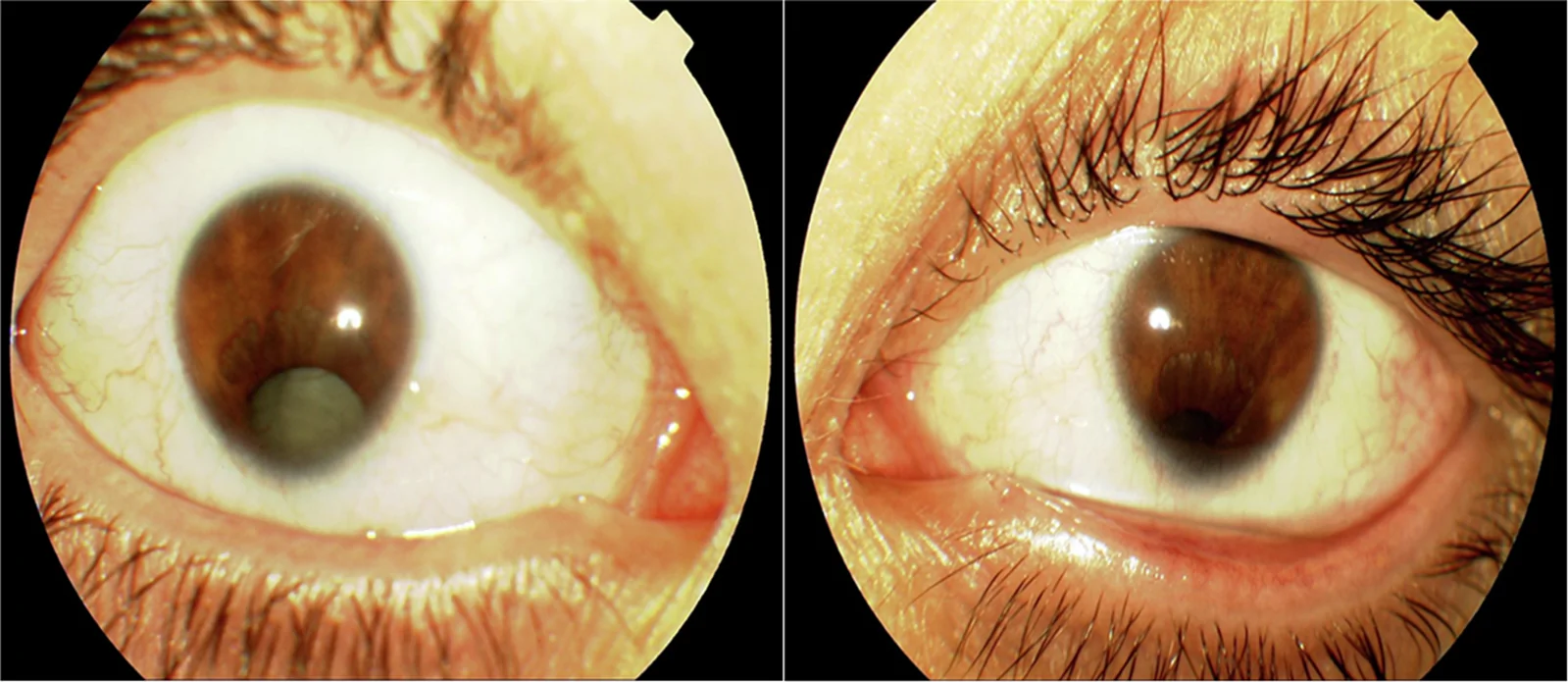
Bilateral coloboma associated with microphthalmos, nystagmus and cataract. Intercalary membrane simulating retinal detachment on B-scan ultrasonography [not shown]. Reproduced from Snead MP. Retinal detachment in childhood. In: Lyons CJ & Lambert SR (eds). Taylor and Hoyt’s Pediatric Ophthalmology and Strabismus. 2023; 6th edn. Elsevier Saunders. pp 613-629.

**Fig. 4:**
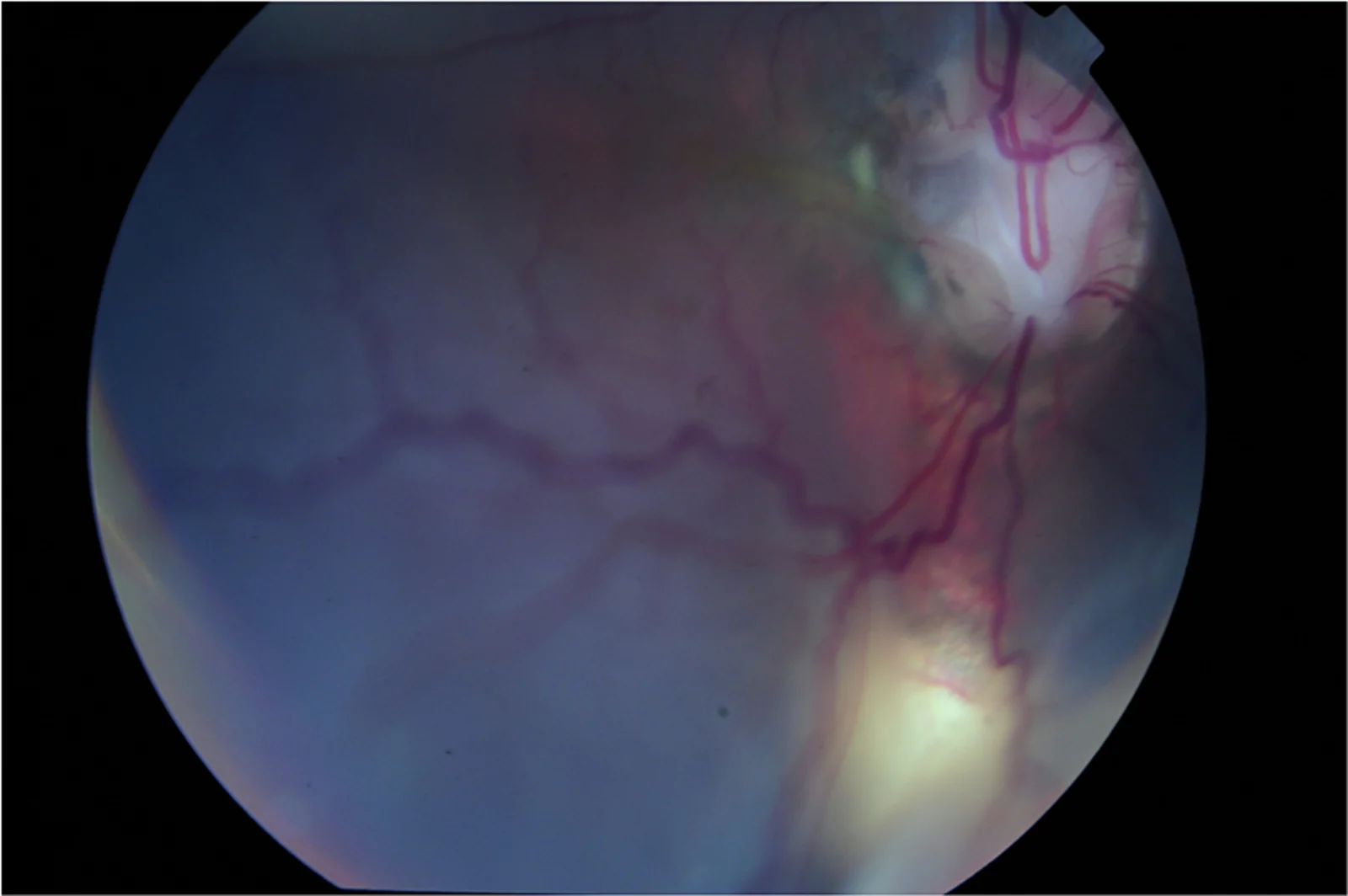
Rhegmatogenous retinal detachment complicating coloboma in a 9-year-old boy. Retinal breaks may be difficult to identify pre-operatively, but visualisation of ‘schlieren’ during vitrectomy can assist localisation. Reproduced from Snead MP. Retinal detachment in childhood. In: Lyons CJ & Lambert SR (eds). Taylor and Hoyt’s Pediatric Ophthalmology and Strabismus. 2023; 6th edn. Elsevier Saunders. pp 613-629.

Limited pupil dilation, nystagmus, microcornea and cataract may all severely compromise any view of the retina and identification of breaks pre-operatively in such cases may be nearly impossible (Fig. 3). Surgery affords the opportunity of halting nystagmus, revising the pupil and dealing with cataract but identification of breaks can still be very difficult against the pale scleral background of the coloboma, particularly for breaks in the ICM. The search may be assisted by the visualisation proteinaceous ‘schlieren-effect’ on internal search (Fig. 4).

**Fig. 5:**
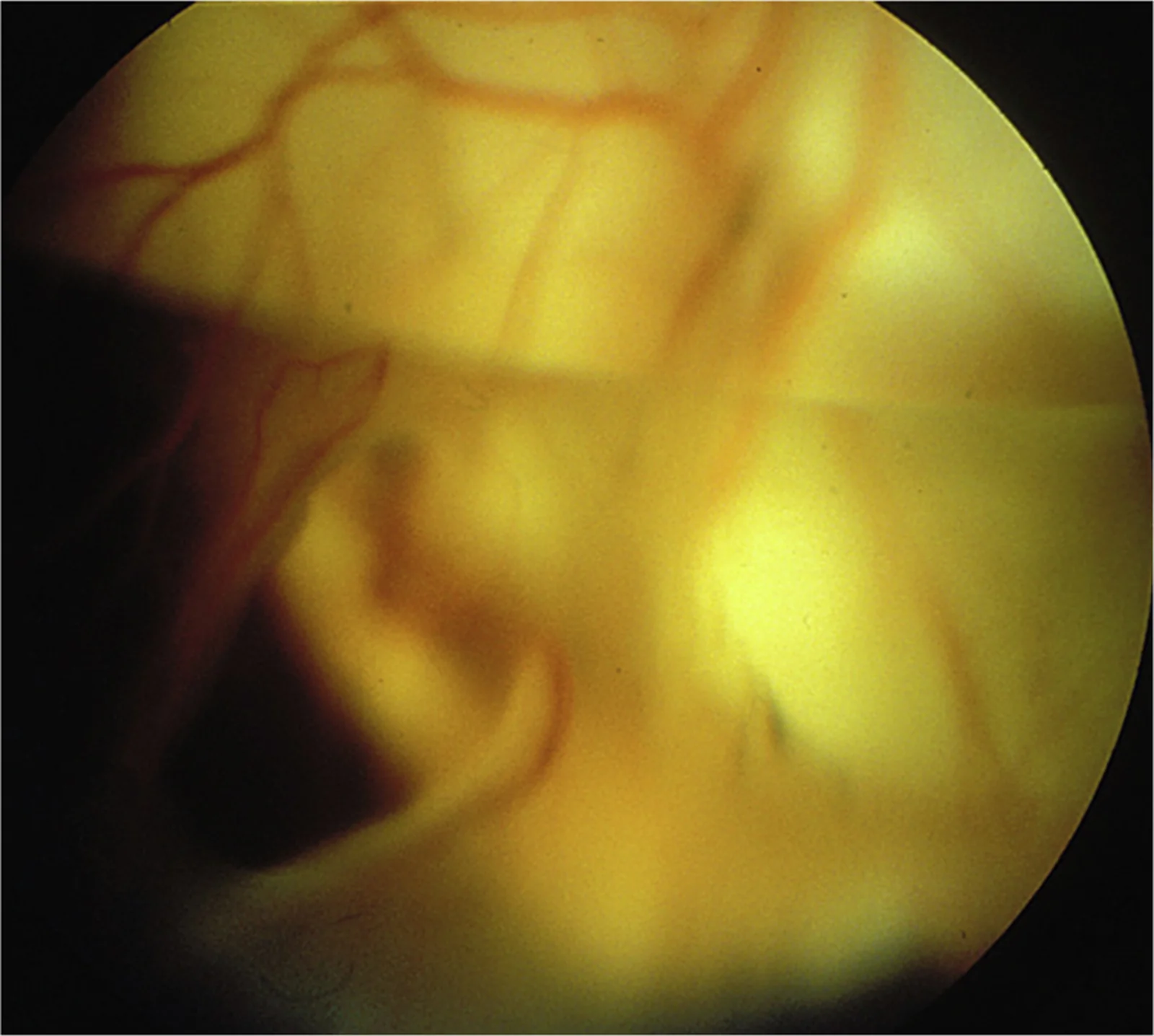
Demonstrates an underfill of silicone oil due to the abnormal configuration of the globe.

**Fig. 6:**
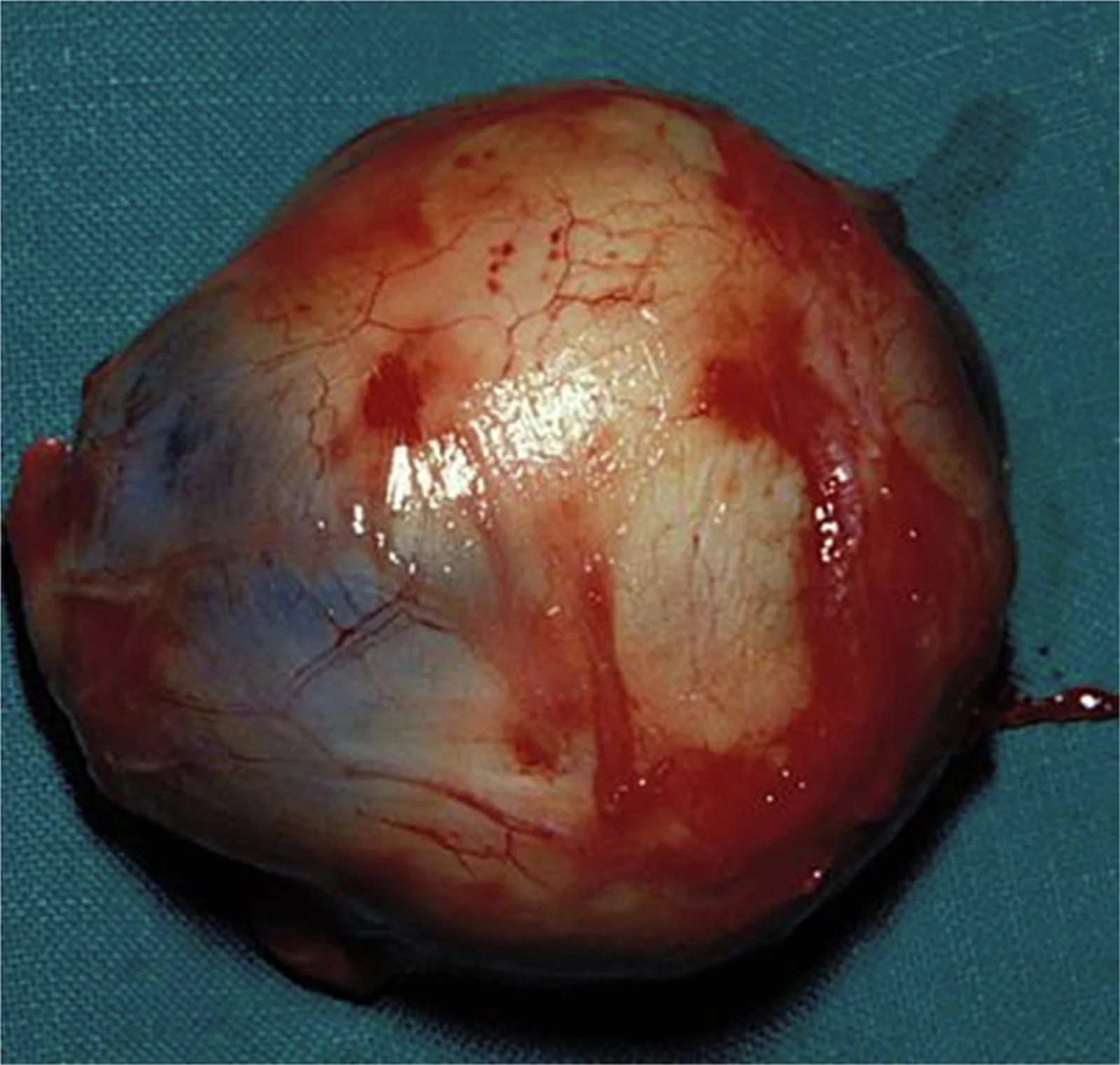
A colobomatous eye with scleral ectasia making scleral buckling an unsafe surgical option.

Retinal detachments complicating choroidal colobomas predominantly affect the inferior fundus and with additional challenges to internal tamponade introduced by the abnormal configuration of the globe (Fig. 5). The associated scleral ectasia may also compromise the safe deployment of an adjunctive scleral buckle to assist in the effective closure of inferior breaks (Fig. 6).

For breaks outside the coloboma, scleral buckling to treat retinal detachment may be deployed in the standard manner, provided the sclera is of adequate quality. For breaks within or close to the coloboma, buckling alone has a low success rate [10, 11] and internal approaches are favored [8, 12, 13]. Whichever approach (or combination of approaches) is adopted, the ultimate goal is to achieve an adequate and permanent adhesion between the neurosensory retina and RPE. Since both RPE and Müller cells are vestigial or absent within the coloboma, effective retinopexy may be impossible to achieve unless applied outside the margin of the coloboma to healthy areas of the retina. In such cases, meticulous identification and treatment of peripheral retinal breaks, along with sealing of the coloboma edge are essential even if an ICM break is not directly visualised. Gopal et al. [12] have suggested that OCT imaging can provide useful adjunctive information to guide retinopexy, particularly if the sinus is close to the point of fixation at the coloboma margin.

Many case series of eyes with posterior coloboma and retinal detachment treated by vitrectomy and long-acting or permanent tamponade report anatomical success rates exceeding 80%, but visual acuities better than 20/400 are achieved in only 35.7–78.4% of eyes [1, 8, 12–17]. Silicone oil removal rates vary between studies, with re-detachment rates post-removal being as high as 27.3% [17]. If the risk/benefit ratio falls in favour of permanent tamponade, then high-viscosity oil should always be deployed to reduce the risk and rate of significant emulsification [18].

## Prophylaxis

Given the complexity of assessment and management of retinal detachment in association with choroidal coloboma, prophylactic retinopexy has been assessed in various studies. The major challenge relates to the need to apply retinopexy adequately to the entire border of the coloboma without compromise to fixation or the papillo-macular bundle. Uhumwangho and Jalali advocated prophylactic laser based on the results of a non-randomised study of 335 eyes in which retinal detachment occurred in 2.9% of the treated group vs 24.1% in the untreated group [19]. Bal et al. conducted a population study of 387 eyes in children and concluded that the risk of retinal detachment in the paediatric population with coloboma was very low, with the risk being only 0.52% per eye and 0.78% per patient [5]. The role of prophylaxis in children is currently unclear and should be decided on an individual case-by-case basis, weighing the risk of potential visual loss from retinal detachment against the risk of collateral damage from prophylactic laser.

### Optic disc pit and retinal detachment

#### Prevalence and pathogenesis

**Fig. 7:**
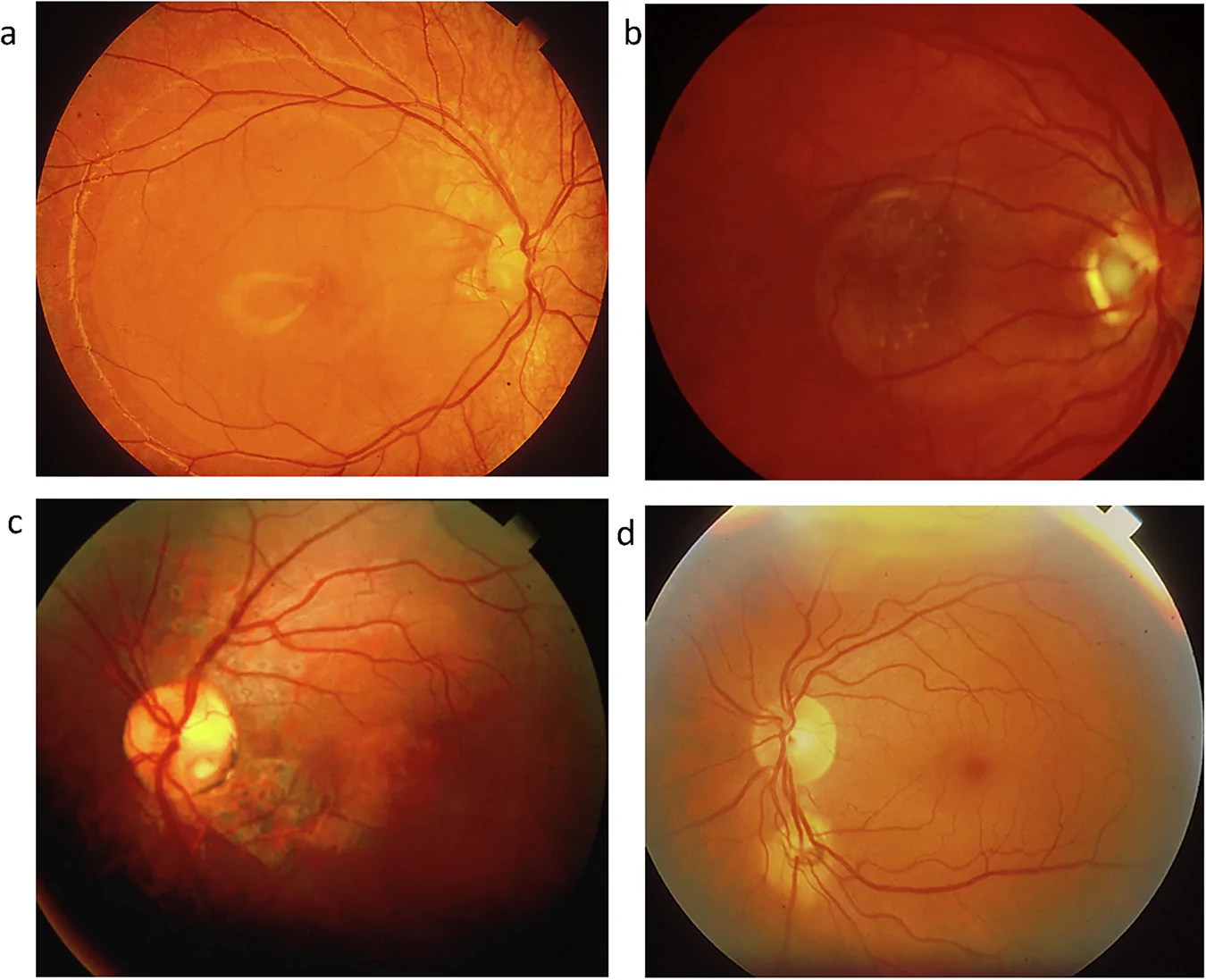
Optic disc pits associated with serous macular detachment. **a**, **b** two examples of optic disc colobomas associated with macular detachment. **c**, **d** child with optic disc pit with serous macular detachment and parent with choroidal coloboma, respectively, illustrating inherited aetiology in some cases. Reproduced from Snead MP. Retinal detachment in childhood. In: Lyons CJ & Lambert SR (eds). Taylor and Hoyt’s Pediatric Ophthalmology and Strabismus. 2023; 6th edn. Elsevier Saunders. pp 613-629.

The prevalence of optic disc pits is estimated to be from 1:11 000 [20] to 19:10 000 [21], and 30-63% [20, 22, 23] of these patients are estimated to suffer from serous macular detachment (Fig. 7). There is no apparent gender predilection and optic disc pits are unilateral in about 85% of cases [24]. Most patients are asymptomatic and the condition is usually discovered incidentally, typically in the second to fourth decade of life. The majority of cases of optic disc pits present as an isolated finding, but the management of retinal detachment can be further complicated when both choroidal coloboma and optic disc dysplasia co-exist within the same eye (Fig. 4). Optic disc pits in association with inherited choroidal coloboma have been reported by several groups [9, 25, 26] (Figs. 7c, d). Although there is little doubt that macular detachment and isolated optic disc pits are causally related, the exact pathogenesis and origin of the subretinal fluid (SRF) have been the subject of much interest and debate.

Fluorescein angiography studies suggest no leakage from either choroidal or retinal circulations or from vessels within the pit, indicating that a vascular source is unlikely [27]. With the advent of OCT imaging, Lincoff et al. [28] and others have suggested a 2-stage process—an initial schisis-like separation of the inner retina allowing subsequent transfer of fluid into the sub-RPE space. However, many reports highlight the presence of SRF without any evidence of associated intraretinal schisis and suggest that any such split may be secondary [29].

Communication between the vitreous cavity and the subretinal space via the optic pit has been demonstrated in collie dogs but not in humans and Irvine et al. described a case of optic nerve sheath fenestration in which (intraocular) gas bubbled out through the nerve fenestration, demonstrating continuity of the vitreous cavity with the perineural space [30].

Current thinking favors either CSF [31–34] or vitreous [22, 35, 36] as the fluid source, with the pit providing a conduit to the sub-retinal space. Regenbogen et al. [34] and later Gass [31] proposed the idea that SRF in optic disc pit maculopathy could be derived from cerebrospinal fluid, but histopathological specimens of optic disc pit associated with macular detachment are understandably rare.

**Fig. 8:**
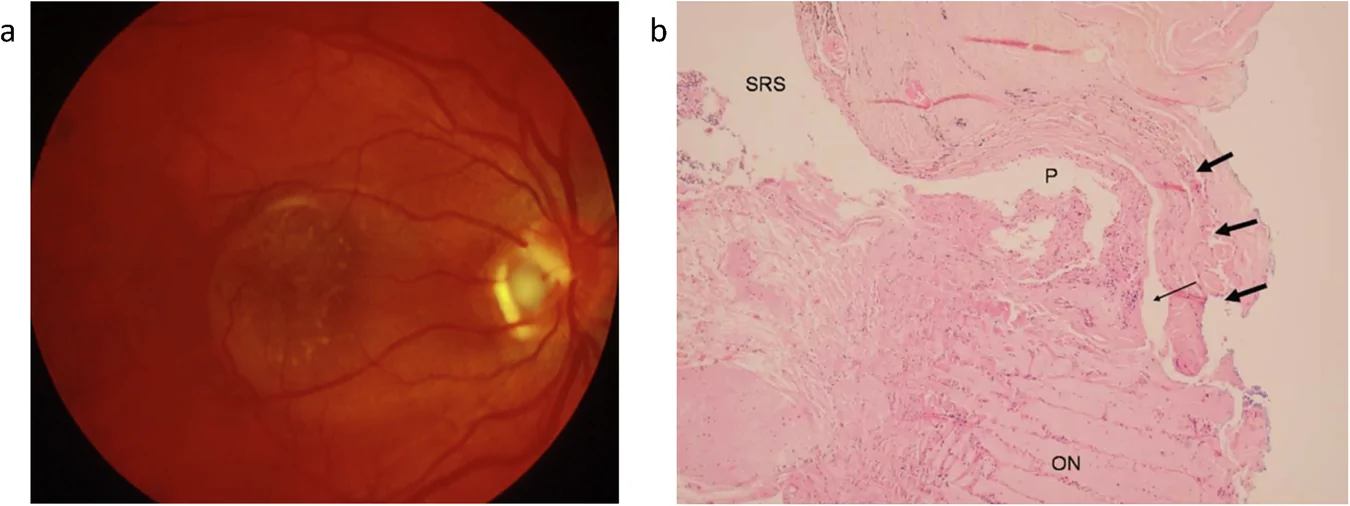
Fundus photograph and histopathology of optic disc pit and associated serous macular detachment. Fundus photograph (**a**) and histology (**b**) of optic disc pit at the inferior margin of optic disc communicated directly with the subretinal space. Note the very thin connective tissue between the subarachnoid space and the pit. ON: section of optic nerve; P: optic disc pit; SRS: subretinal space; thick black arrows: pia-lined subarachnoid space; thin black arrow: either artefactual split or extension of the subarachnoid space. Reproduced from Poulson AV, Snead DRJ, Jacobs PM, Ahmad N, Snead MP. Intraocular surgery for optic nerve disorders. Eye. 2004 Nov;18(11):1056–65.

Gass [31] reported the histopathology in two eyes showing herniation of dysplastic retina into a collagen-lined pit extending posteriorly through a defect in the lamina cribrosa into the subarachnoid space. He theorised that CSF access to the subretinal space was more likely than vitreous since it had no epithelial barrier to cross. This theory is supported by Poulson et al. [37], who also reported on the histopathology of a case where the optic disc pit lying at the inferior margin of the optic disc communicated directly with the fluid in the subretinal space. (Fig. 8) The connective tissue between the subarachnoid space and the pit was extremely thin and although no direct communication was identified, it supported the Regenbogen theory that CSF could transudate across the thin collagenous membrane to the pit.

Further support for the CSF origin of the SRF in optic disc pit maculopathy is provided by Makdoumi et al. [38], who reported beta-trace protein levels in the SRF of four patients with a macular detachment associated with optic disc pit, albeit at lower levels than are typically found in CSF. Beta-trace protein has high concentrations in the CSF and has been used as a surrogate for confirming the presence of CSF leakage into the nasal cavity [38].

#### Natural history and management

Natural history studies of optic disc pit-associated retinal detachment generally suggest a poor prognosis, although spontaneous resolution of the macular detachment has been reported to occur in up to 25% of cases [27]. There are also numerous reports [39–46] of stable or spontaneous resolution. Bloch et al. found that out of the 52 patients that were managed conservatively, 77% maintained 6/12 or better [47], concluding it was best to delay intervention unless visual deterioration occurred. By contrast, in another study of 15 eyes with an average follow-up of 9 years, 12 out of 15 patients had visual acuities of 20/200 or worse at the last examination [48] and in another study of 20 patients with more than 1-year follow-up, 55% had visual acuity of 6/30 or worse [22]. Therefore, for symptomatic patients who do not show evidence of resolution, medical or surgical intervention is usually offered.

Al-Moujahed et al. reported a case treated with topical dorzolamide and improved visual acuity from 20/30 to 20/20 with resolution of intraretinal fluid over a 2-year period [49], but in such isolated reports it is not possible to determine whether the resolution of the serous maculopathy was part of the natural history or as a result of the treatment.

Prakash et al. reported oral acetazolamide being used successfully in a case of morning glory syndrome [50] and Osigian et al. reported success in a patient who was commenced on oral acetazolamide as part of a weight loss regime after re-occurrence of subretinal fluid after initial success with vitrectomy, inner limiting membrane (ILM) peel, juxtapapillary endolaser and gas tamponade [51]. The rationale for the use of carbonic anhydrase inhibitors is to induce subretinal fluid resorption via acidification of the subretinal space [52], as well as their additional role in lowering intracranial pressure (see below) [51]. This mechanism is in concordance with the pressure gradient theory in serous macular detachment and optic disc pit [29]. However, evidence is limited and the subretinal fluid may reoccur after discontinuation of treatment [50]. Oral steroids have also been deployed as treatment, but both the efficacy and rationale remain unestablished [53, 54].

**Fig. 9:**
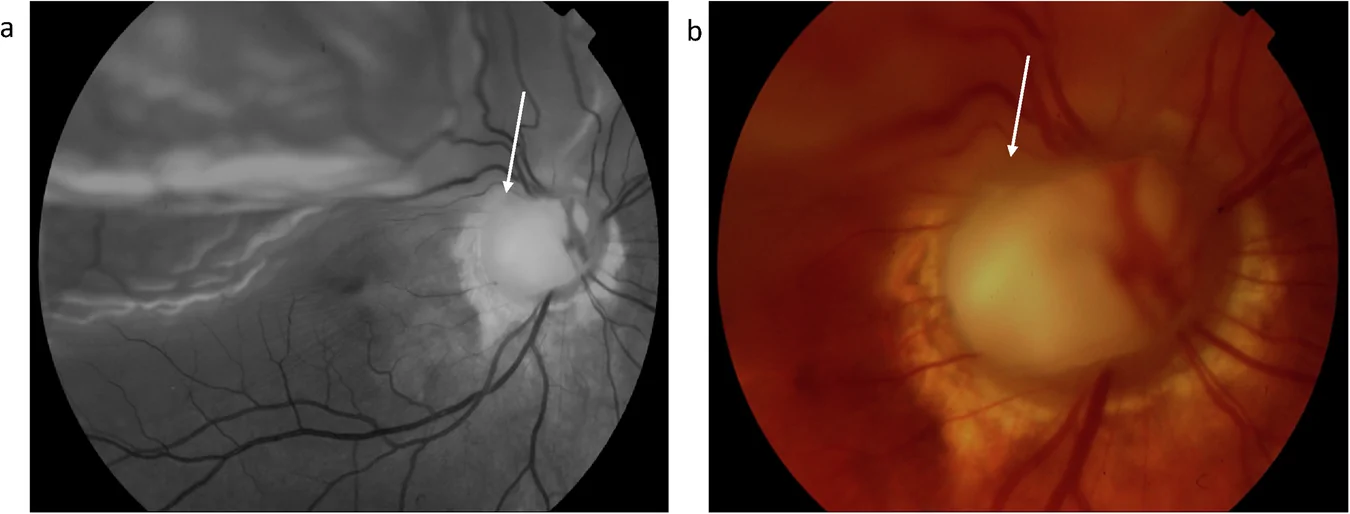
Red free and colour photograph of optic disc pit and associated retinal detachment. The white arrow points to the fluid spilling over the optic disc margin and into the pit, demonstrating the bullous superior retinal detachment linked to sinus of fluid deep within optic disc pit. **a** red-free photo; **b** coloured fundus photo of higher magnification.

Irrespective of whether the subretinal fluid derives from CSF transudate or the vitreous cavity, consensus favors the pit as the final conduit between origin and subretinal space; therefore, surgical approaches to treatment have principally focused on obliterating the communicating sinus between the two (Fig. 9).

**Fig. 10:**
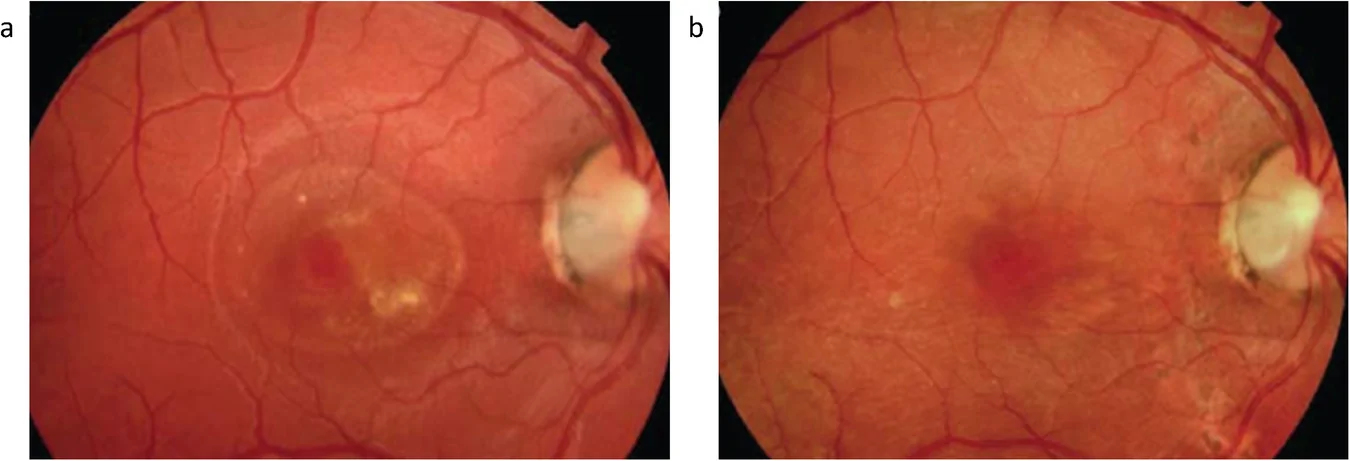
Vitrectomy, gas tamponade and endolaser for optic disc pit maculopathy, pre- and post-operative images. **a** pre-surgery VA was 6/36. **b** post-surgery VA was 6/12. Thermal injury to the papillo-macular bundle can be minimised by applying laser prior to fluid/ gas exchange.

Early attempts using a juxta-papillary laser to create a chorioretinal scar as a barrier to fluid migration from the optic nerve to the macula met with mixed results [55–58]. Current literature suggests that laser photocoagulation alone is often unsuccessful, although its benefits in combination with pars plana vitrectomy (PPV) are less clearly defined [31, 55, 59–61]. The risk of juxtapapillary photocoagulation damaging the papillo-macular nerve bundle causing iatrogenic scotomas [57, 62, 63] can be mitigated by applying the laser before the fluid/gas exchange phase of the operation (Fig. 10) (ref. 61).

Pars plana vitrectomy and gas tamponade are currently the most favoured surgical approach, with various adjuncts including endolaser, ILM peeling, ILM flap and even the use of silicone punctal plug [64, 65]. There is no current consensus on which (if any) of such adjuncts offer any more favourable outcomes. A systematic review analysing 59 studies found PPV alone achieved complete anatomic success in almost 90% of cases and visual improvement in all of these [66], although across all uncontrolled studies, the use of gas was associated with a slightly lower rate of both anatomical success and visual improvement [66]. ILM peeling and attempts to occlude the pit with an ILM plug were not associated with any additional benefit and this is perhaps not surprising given the likely CSF origin of the fluid.

Results differ slightly in the large EVRS multicentre study, where vitrectomy with gas tamponade was shown to be associated with a higher likelihood of better anatomical and functional outcomes [59]. Both studies concluded that ILM peeling during PPV provided no additional benefit and the EVRS study also concluded that addition of juxta-papillary laser was of no additional benefit [59]. The EVRS study found that 15% of cases required retreatment, but of these, there was only anatomical improvement without functional improvement [59]. In contrast, the recurrence rate of optic disc maculopathy after PPV was only found to be 2% in the 2021 meta-analysis [66].

The use of gas tamponade alone may serve as a temporary barrier between the vitreous cavity and optic disc pit and may further displace any intraretinal fluid to the outer layer detachment, enhancing fluid resorption and reattachment [59, 66, 67]. The authors’ experience is to favour vitrectomy and gas tamponade with combined endolaser, with the rationale that this approach is most likely to afford a permanent occlusion of communication between the pit and sub-retinal space (Table 2).

**Table 2:** Case series of patients with retinal detachment secondary to optic disc pit at Cambridge University Hospital.

Age (years)	Sex	Presenting VA	Treatment	Anatomical outcome	Final VA	Duration of follow-up (months)
20	F	6/18	Vitrectomy, gas, laser	Attached	6/9	6
9	M	6/60	Vitrectomy, gas, laser	Attached	3/60	29
27	F	CF	Vitrectomy, gas, laser	Attached	6/18	30
16	M	6/18	Observation	Attached (macula)	6/18	24
19	F	6/36	Vitrectomy, gas, laser	Attached	6/12	6
34	M	6/24	Observation	Detached	Unknown	Patient died from septicaemia
14	F	6/6	Observation	Attached	6/6	96

The efficacy of adjunctive ILM peeling (with or without the ILM flap to ‘plug’ the disc pit) remains unproven. In the EVRS study, 62% of the eyes treated with vitrectomy ILM peeling showed no additional anatomical or functional advantage and adjunctive ILM peeling was associated with an apparently higher risk of secondary macular hole, although the numbers were too small to reach statistical significance [59]. The meta-analysis by Meng et al. also found ILM peeling to be of no additional benefit [66].

## Coloboma of the crystalline lens and retinal detachment

Ciliary body dysgenesis leading to regional loss of zonular fibres is not strictly a true coloboma, but it is nevertheless well recognised in association with retinal detachment and particularly giant retinal tear [68–70].

Although the vitreous body is conventionally considered an inert, transparent body playing a purely structural role, this concept eclipses its other vital role in cell signaling homeostasis during embryogenesis. The major structural component of the vitreous is type II collagen, but this is expressed in two isoforms: a long form (type IIa) in the vitreous and a short form (type IIb) in articular cartilage. The long form contains an additional domain in N-propeptide that binds and regulates members of the TGF-β and Bone Morphogenetic Protein (BMP) superfamily, which in turn transduce intracellular signaling controlling growth, especially of the ciliary body, iris and lens [71].

**Fig. 11:**
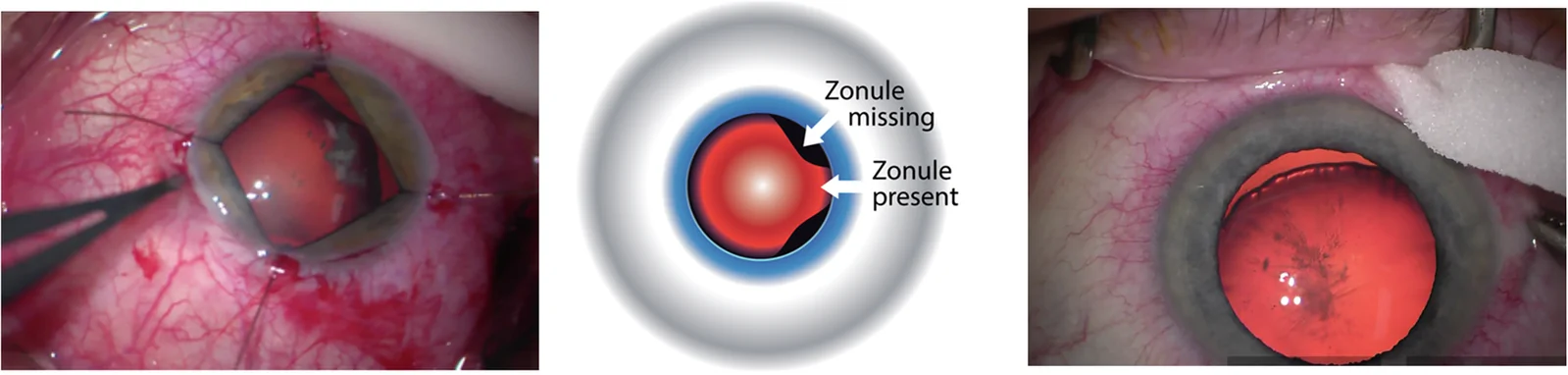
Giant retinal tear, retinal detachment, and zonule/ciliary dysgenesis are associated with both Stickler syndrome and familial or bilateral retinal detachment. Figure shows two different eyes with Type 1 Stickler syndrome with regional dysgenesis of ciliary processes and absent zonular fibres with a schematic representing the same. Reproduced from Snead MP, Lovicu FJ, Nixon TRW, Richards AJ, Martin H. Pathobiology of the crystalline lens in Stickler syndrome. Progress in Retinal and Eye Research. 2024 Nov 1;103:101304.

A murine col2a1 knock-out model of *COL2A1* haploinsufficiency designed to mimic the absence of this binding domain on one allele exhibits repressed development of ciliary processes [72] and such defects are frequently seen clinically in Stickler syndrome (Fig. 11) as well as in other patients presenting with rhegmatogenous retinal detachment.

A parallel study of UK patients with either bilateral or familial retinal detachment by Spicket et al. [73] identified a significant association in affected patients. They were homozygous for a relatively common variant affecting transcription of the same region of the gene for type II collagen that binds the growth factors TGFβ1 and BMP-2.

From a practical management perspective, this may manifest as an immediate transfer of gas to the anterior chamber during fluid/gas exchange at vitrectomy surgery (as well as the obvious zonular instability during any subsequent post-vitrectomy cataract surgery). The risk of gas migrating perioperatively into the anterior chamber may be mitigated by retaining a barrier of gel anteriorly in the retrolental space, although in conditions where the vitreous development is congenitally arrested, it should be anticipated that this is less likely to be possible.

## Conclusion

Retinal detachment complicating choroidal coloboma, optic disc pits, or associated with lens coloboma is rare and challenging to manage due to multiple factors. For retinal detachment associated with choroidal coloboma, type IIA and type IIB retinal detachment are particularly challenging due to poor pre- and per-operative visualisation of retinal tears, which makes adequate and permanent adhesion between the neurosensory retina and RPE difficult to achieve. Internal approaches are favoured for type IIA and IIB detachments, while scleral buckling may be deployed for type I detachments, provided the sclera is of adequate quality.

For optic disc pit-associated retinal detachments, the key to management is to obliterate the communicating sinus to the subfoveal space. This usually involves pars plana vitrectomy and gas tamponade. For those favouring adjunctive juxtapapillary endolaser, application prior to fluid/gas exchange mitigates the risk of collateral thermal injury to the papillo-macular bundle.

In retinal detachments associated with lens coloboma, the principal challenge is the immediate transfer of gas to the anterior chamber during fluid/gas exchange, with its attendant problems of fundus visualisation at a critical juncture, often before retinopexy has been applied. Due to the rarity of these conditions, consensus in management is difficult to establish and care should be individualised on a case-by-case basis due to the variability in presentation.
